# Outbreak of feline tuberculosis caused by *Mycobacterium bovis* in a German household, a possible domestic zoonosis

**DOI:** 10.1177/10406387261447251

**Published:** 2026-05-14

**Authors:** Hande Peters, Luise Kaspers, Hanka Brangsch, Elisabeth M. Liebler-Tenorio, Claudia Bunzenthal, Ann-Kathrin Kühling, Annette Kuczka, Miriam Golestan, Sandra Seifert, Stefanie A. Barth

**Affiliations:** Chemical and Veterinary Investigations Office Rhein-Ruhr-Wupper, Krefeld, Germany; Chemical and Veterinary Investigations Office Rhein-Ruhr-Wupper, Krefeld, Germany; Institute of Bacterial Infections and Zoonoses, Friedrich-Loeffler-Institut/Federal Research Institute for Animal Health (FLI), Jena, Germany; (Brangsch); Institute of Molecular Pathogenesis, Friedrich-Loeffler-Institut/Federal Research Institute for Animal Health (FLI), Jena, Germany; Chemical and Veterinary Investigations Office Rhein-Ruhr-Wupper, Krefeld, Germany; Chemical and Veterinary Investigations Office Rhein-Ruhr-Wupper, Krefeld, Germany; Chemical and Veterinary Investigations Office Rhein-Ruhr-Wupper, Krefeld, Germany; Tierarztpraxis Fell & Feder, Köln, Germany; Stadt Köln, Umwelt- und Verbraucherschutzamt, Lebensmittelüberwachung und Veterinärdienste, Köln, Germany; Institute of Molecular Pathogenesis, Friedrich-Loeffler-Institut/Federal Research Institute for Animal Health (FLI), Jena, Germany; National Reference Laboratory for Bovine Tuberculosis, Jena, Germany

**Keywords:** cats, cgSNP, dyspnea, *Mycobacterium bovis*, serology, tuberculosis, WGS

## Abstract

Infections with *Mycobacterium bovis* can lead to clinical tuberculosis in many mammals. Here, we describe *M. bovis* infections in 4 of 7 cats from 1 household in Germany. These cats had respiratory disorders at intervals of several months. Two of 4 euthanized animals (cats 3 and 4) were submitted for postmortem examination; lungs failed to collapse and were firm in both cats. In cat 4, a severely enlarged pulmonary lymph node was found, as well as small white granulomas in the spleen, liver, and kidney. Bacterial cultivation identified *M. bovis* spoligotype SB0120 as the causative pathogen in cats 3 and 4. Molecular genetic fine typing revealed that this genotype had not been reported previously in animals or humans in Germany. Serum from cat 4 shortly before euthanasia, and from cats 5–7, were tested for TB-specific antibodies by ELISA. Cat 4 was strongly positive. In contrast, cats 5–7 were negative and remained negative 4 mo later. Based on our case series, tuberculosis should be considered as a differential diagnosis in pet animals, even in countries that are officially free of the disease. A lack of awareness about tuberculosis could increase the risk of zoonotic infections with *M. bovis*—for both owners and other animals living in affected households.

Tuberculosis (**TB**) in animals is a zoonosis caused by members of the *Mycobacterium tuberculosis* complex (**MTC**), in particular *M. bovis* and *M. caprae*, and rarely *M. tuberculosis, M. microti*, or other members of the MTC. Mammalian tuberculosis is a World Organisation for Animal Health (WOAH)-listed disease, and countries are required to report its occurrence in bovid, cervid, caprid, and camelid hosts.^60^ The main reservoirs of *M. bovis* and *M. caprae* are cattle and small ruminants, but wildlife reservoirs include badgers in Great Britain, red deer in the Alps, wild boar in the Iberian Peninsula, and bison in Poland.^6,18,30,38^

Feline tuberculosis is a well-known disease. Case descriptions exist from as early as the 1950s in Switzerland^52^ and the 1980s in New Zealand.^14^ The number of feline TB cases has decreased in concert with the decreasing numbers of bovine TB cases. Hence, awareness of the disease by veterinarians and owners has declined. In cats, 2 main groups of MTC infections share some of their clinical signs but differ in lesions, causative pathogen, and mean age of affected cats.^21,32^ Cases of the first group, with cutaneous nodules and lymphadenopathy, are infected by *M. microti.*^39^ The reservoir host for *M. microti* is the wild field vole^10^ and most infections occur via scratches in vole-hunting cats. The second group of feline MTC infections is caused by *M. bovis* or *M. caprae.* These systemic infections originate in the respiratory or digestive tracts and occur predominantly in regions where TB is endemic in cattle.^4,15,21^

Rarely, MTC infections are reported from officially tuberculosis-free (**OTF**) countries.^13^ Potential routes of infection include consumption of unpasteurized milk or direct contact with infected cattle in TB-affected cattle farms.^9^ Larger outbreaks associated with the consumption of commercial raw meat have been reported in the United Kingdom.^35,36^ Sometimes, the route of infection remains unclear (e.g., when animals have been imported from other countries).^3,11^ Infected cats often have reduced body condition, cachexia, lymphadenopathy, or respiratory disorders.^35^ Atypical but recognized presentations of ocular and joint infection occur in association with *M. microti* and *M. bovis* infections.^31,51^ The mean age of cats affected by *M. microti* is ~8 y. Cats infected with *M. bovis* are significantly younger, with a mean of ~3.5 y.^21^

All members of the MTC are considered zoonotic pathogens.^16^ Therefore, an infectious risk for humans exists, especially with pet animals, which often live in close contact with their owners.^5,13,27,34,37^ Here, we describe an outbreak of feline TB in an urban household in Germany that included 7 cats. Six rescue animals had been transferred from Turkey to Germany by a private pet agency; 4 of these 6 cats had respiratory disorders.

## Material and methods

### Animals and history

**Figure 1.**
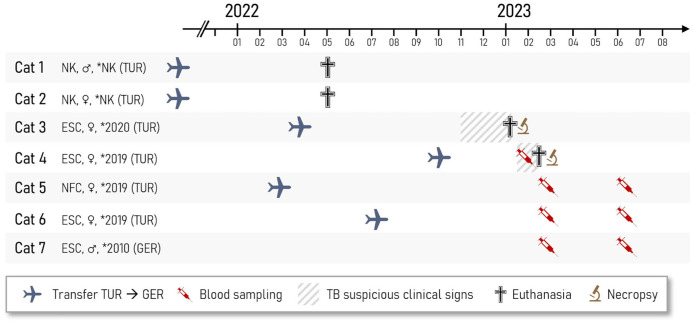
Feline tuberculosis in a German household. Timeline for the available information on breed, sex, birthdate, transfer to Germany, duration of clinical signs, blood sampling, and death of the cats. ESC = European Shorthair cat; GER = Germany; NFC = Norwegian Forest cat; NK = not known; TUR = Turkey; * = year of birth.

Seven cats were kept in an urban household on the outskirts of a large German city. Six of these cats were imported from Turkey by a private pet agency (**Fig. 1**). No clinical history was available. The cats were regularly vaccinated against feline calicivirus, feline herpesvirus, feline leukemia virus, and feline parvovirus.

All 7 animals had access to their owner’s house and garden, but did not leave the property. There were no livestock farms nearby or accessible to the cats. In Germany, the animals were fed a commercial diet (dry and wet food) and had no access to unpasteurized raw milk or raw meat.

The examinations performed and results were part of the diagnostic approach and follow-up by the official authorities (local veterinary office and public health department in North Rhine-Westphalia). Therefore, no ethical approval was required.

### Postmortem examination

The postmortem examinations of cats 3 and 4 were conducted by pathologists at the Chemical and Veterinary Investigations Office Rhein-Ruhr-Wupper (CVUA-RRW; Krefeld, Germany) following internal standardized operating procedures. Samples of various organs were taken for histologic analysis. Additionally, samples of the lung of cat 3 and tracheobronchial lymph node of cat 4 were examined via bacterial culture and molecular testing.

For histologic examination of tissue sections, H&E, Grocott (fungi), Azan (connective tissue), and Ziehl–Neelsen or Kinyon (acid-fast bacteria) stains were applied according to standard protocols. Immunohistochemistry (IHC) was performed as an ancillary test with a polyclonal antibody specific for *M. bovis* (BYT-ORB100411; Biozol). A negative control using a polyclonal antibody against an unrelated antigen as the primary antibody was included for each section. Bovine skeletal muscle tissue was spiked with *M. bovis* to serve as the positive control.

### Microbiologic and molecular genetic detection of mycobacteria

#### Cultivation of tissue samples

Tissue samples from the lung of cats 3 and 4 and tracheobronchial lymph nodes of cat 4 were homogenized and decontaminated with N-acetyl-L-cysteine-NaOH (final concentration 1%, 25 min, shaking). After 2 washing steps, 1 part of the suspension was used for DNA extraction and the other part was cultured on Stonebrink agar slants (supplemented with PACT [polymyxin, amphotericin, carbenicillin, trimethoprim]; Becton Dickinson), Herrold egg-yolk agar,^59^ Middlebrook 7H11 agar slants supplemented with vancomycin and amphotericin, and Middlebrook 7H10 agar slants (all in-house), as well as in MGIT/PANTA/OADC broth (Becton Dickinson). Cultures were incubated at 37 ± 2°C for up to 12 wk and examined weekly for suspicious colonies.

#### Real-time PCR from tissue samples

DNA was extracted from homogenized tissues (DNeasy blood and tissue kit; Qiagen) and tested for MTC-specific DNA according to the German official manual of diagnostic procedures for epizootics.^19^ In brief, 2 real-time PCR (rtPCR) assays targeting the MTC-specific IS*1081* and a putative helicase gene, respectively (each in combination with the amplification of a fragment of the host’s β-actin gene), were used (QuantiTect multiplex PCR NoROX kit; Qiagen).

### Molecular characterization of the isolates

#### End-point PCR assays for species identification

Species identification of the MTC isolates was done using end-point PCR assays targeting regions of difference (**RDs**). In detail, the presence or absence of RD4 and RD12,^57^ as well as RD9,^8^ was checked according to published protocols.

#### Spoligotyping

Spoligotyping was performed using a DNA microarray format (ArrayStrip platform; Alere Technologies) according to the manufacturer’s instructions^46^ and using the mbovis.org database to identify the profile.^49^

#### Mycobacterial interspersed repetitive unit-variable number of tandem repeats (MIRU-VNTR)

MIRU-VNTR typing of the *M. bovis* strains was carried out using an in-house technique,^7^ according to published protocols^53^ with slight modifications.^20,48^ Overall, we tested 26 VNTR loci: VNTR154 (MIRU-2), VNTR424 (Mtub04), VNTR577 (ETR-C), VNTR580 (MIRU-4, ETR-D), VNTR802 (MIRU-40), VNTR960 (MIRU-10), VNTR1644 (MIRU-16), VNTR1955 (Mtub21), VNTR2059 (MIRU-20), VNTR2163b (QUB-11b), VNTR2165 (ETR-A), VNTR2347 (Mtub29), VNTR2401 (Mtub30), VNTR2461 (ETR-B), VNTR2531 (MIRU-23), VNTR2687 (MIRU-24), VNTR2996 (MIRU-26), VNTR3007 (MIRU-27, QUB-5), VNTR3171 (Mtub34), VNTR3192 (MIRU-31, ETR-E), VNTR3690 (Mtub39), VNTR4052 (QUB-26), VNTR4156 (QUB-4156), VNTR4348 (MIRU-39), VNTR2163a (QUB-11a), and VNTR3232 (QUB-3232).

#### Whole-genome sequencing

For DNA preparation, isolates were grown on Coletsos agar (Artelt-Enclit) for at least 6 wk. The DNA was extracted using the cetyltrimethylammonium bromide method^55^ with additional RNase A digestion and washing steps. DNA was sent to a commercial sequencing service (Eurofins Genomics) to generate whole-genome sequencing (WGS) data using NovaSeq technology (Illumina). The raw sequencing data of both isolates were deposited in the European Nucleotide Archive (ENA) under project PRJEB104949.

#### Bioinformatic analysis of WGS data

The raw sequence data were uploaded to the TB profiler pipeline (https://tbdr.lshtm.ac.uk/) to determine the spoligotype, affiliation to known genetic lineages, as well as prediction of anti-tuberculosis drug resistances (accession date 2024 Aug 16).^12,40^ Core genome SNP calling was conducted (Snippy v.4.6.0, https://github.com/tseemann/snippy) with *M. bovis* strain AF2122/97 (GCF_000195835.3) as reference. Based on the core genome single-nucleotide polymorphism (**cgSNP**) alignment, SNP distances were determined (snp-dists v.0.8.2, https://github.com/tseemann/snp-dists). A maximum-likelihood tree was generated with RAxML v.8.2.12,^50^ applying the GTRCAT model with the -V option and Lewis ascertainment bias correction, as recommended in the RAxML manual. The tree was visualized using Microreact (https://microreact.org/).^2^ For comparison, raw sequence data of German *M. bovis* strains of spoligotype SB0120 isolated from different hosts^28^ were downloaded from the NCBI Short Read Archive and included in the cgSNP analysis.

### Serology

One serum sample was available from cat 4 ~2 d before euthanasia. Additionally, sera from cats 5–7 were collected in parallel to cat 4 as well as 4 mo later. The sera were analyzed (Ingezim DR ELISA, 1:25 diluted; Gold Standard Diagnostics) according to the manufacturer’s instructions. The cutoff was determined with the kit internal positive and negative controls.

## Results

### Case descriptions

#### Cats 1 and 2

The cat owners reported that cats 1 and 2, both imported from Turkey, fell ill in May 2022 with severe respiratory signs similar to those of cats 3 and 4. Cats 1 and 2 were euthanized by local veterinarians given their deteriorating general condition. No further examinations were carried out on these cats.

#### Cat 3

**Figure 2.**
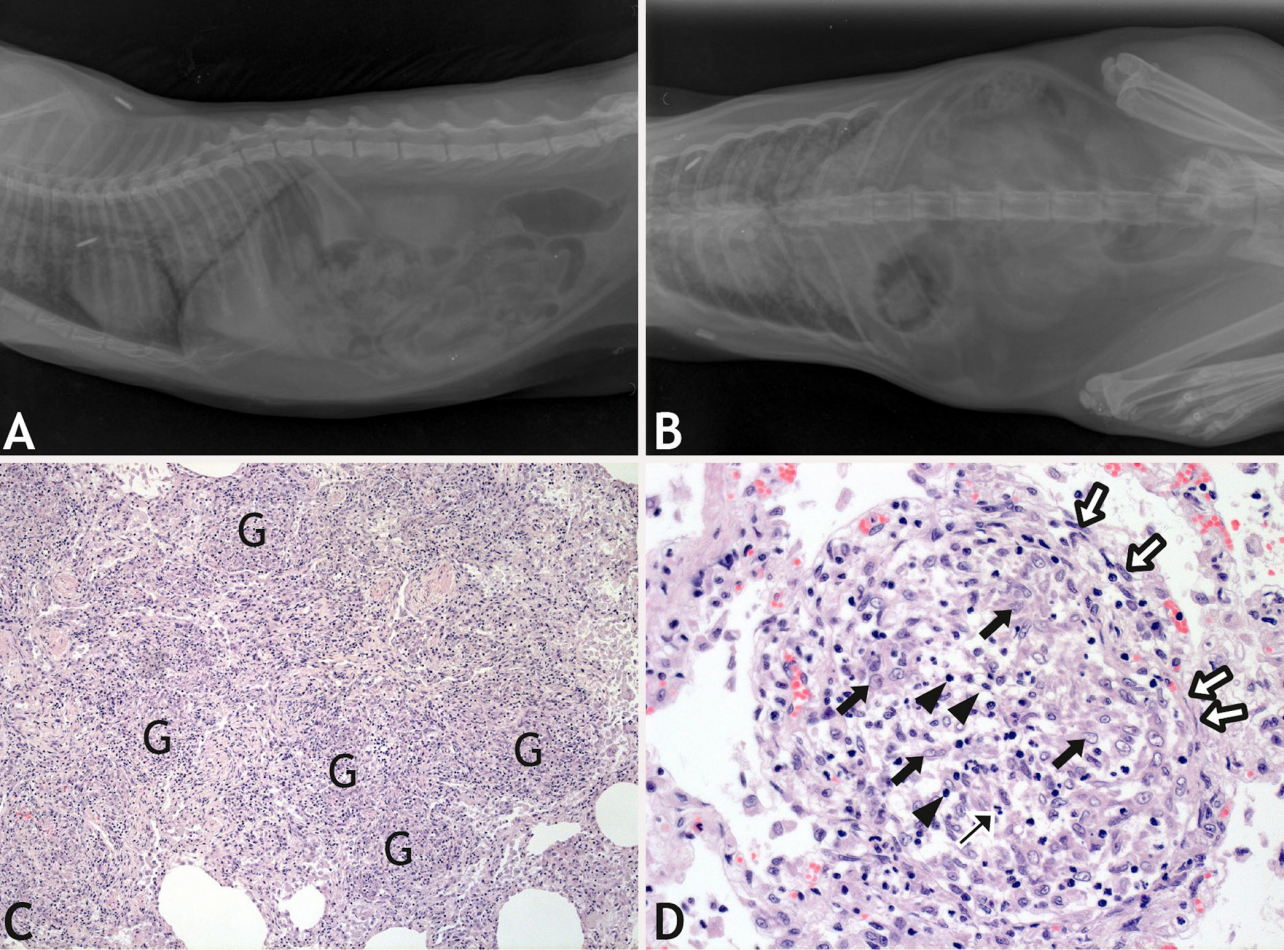
Tuberculosis in cat 3 in a German household. **A**. Left-lateral and **B**. dorsoventral radiographs of the lung taken in Nov 2022, with consolidated lung tissue. **C.** Histology of the consolidated lung with numerous confluent granulomas (G). H&E. **D.** A granuloma consisting of a mixed infiltrate of epithelioid cells (thick arrows), macrophages, lymphocytes (arrowheads), and a few neutrophils (thin arrow). The granuloma is demarcated by a thin layer of fibrous connective tissue (white arrows). H&E.

Cat 3 was a 3-y-old, female European Shorthair cat that was initially in very good nutritional condition. Starting in November 2022, the cat was dyspneic, had a reduced appetite, and lost weight. The local veterinarian performed a radiographic examination, which revealed compacted lung tissue with condensation of the lung parenchyma (**Fig. 2A, 2B**). A presumptive diagnosis was lung mycosis or parasitosis. A CBC indicated mild anemia and inflammation, with lymphocytosis. A biochemistry panel identified a decreased albumin:globulin ratio (**Table 1**).

**Table 1.** Hematology and serum biochemistry values outside their RIs for cat 3.

Measurand	RI	Date of blood sampling	
		Jun 2022	Nov 2022
WBCs, ×10^9^/L	3.9–12.5	20.7	16
Hemoglobin, g/L	108–169	93	101
Hematocrit	0.36–0.56	0.32	0.34
Mean corpuscular volume, fL	42–57	41	40
Mean corpuscular hemoglobin, pg	13–17	12	12
Lymphocytes, ×10^9^/L	1–4.9	9.3	7.0
Monocytes, ×10^9^/L	0–0.6	0	0.6
Eosinophils, ×10^9^/L	0–1.3	2.1	0.6
Aspartate aminotransferase, μkat/L	0–0.78	0.45	1.14
Cholesterol, mmol/L	2.7–9	2.2	1.9
Albumin:globulin ratio	0.5–1	0.5	0.4
Albumin, g/L	28–41	26	26
Globulin, g/L	32–58	57	61

The body condition declined rapidly over the following weeks, and, in mid-January 2023, the cat was euthanized for animal welfare reasons. The dominant alterations at autopsy were marked anemia and consolidated lungs. The tracheobronchial lymph nodes were not detectable. Liver and spleen were slightly enlarged. The liver, spleen, and kidneys were hyperemic. The right cardiac ventricle was slightly dilated.

Histologically, granulomas in the lung were surrounded by diffuse mixed-cell infiltrates; alveolar histiocytosis was moderate to severe (**Fig. 2C**). Granulomas were demarcated extensively by fibrous connective tissue (**Fig. 2D**). Neither bacteria nor fungi were detected by special stains, and immunohistochemistry for *M. bovis* was negative.

MTC-specific DNA was detected by rtPCR testing of the lung tissue. Subsequently, *M. bovis* was identified by culture.

#### Cat 4

**Figure 3.**
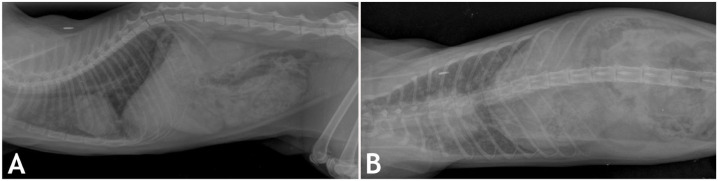
Tuberculosis in cat 4 in a German household. **A**. Left-lateral and **B**. dorsoventral radiograph of the lung taken in Nov 2022, with consolidation of the lung tissue in the cranial lung field.

Cat 4 was an ~4-y-old, female European Shorthair cat. The radiographic examination performed in November 2022 revealed consolidation of the left cranial lung lobe (**Fig. 3A, 3B**), but only mild clinical signs of respiratory disease. In January 2023, more severe respiratory abnormalities developed. Given the diagnosis of TB received for cat 3 and a daily decline in its health status, cat 4 was euthanized for animal welfare reasons and submitted for autopsy.

**Figure 4.**
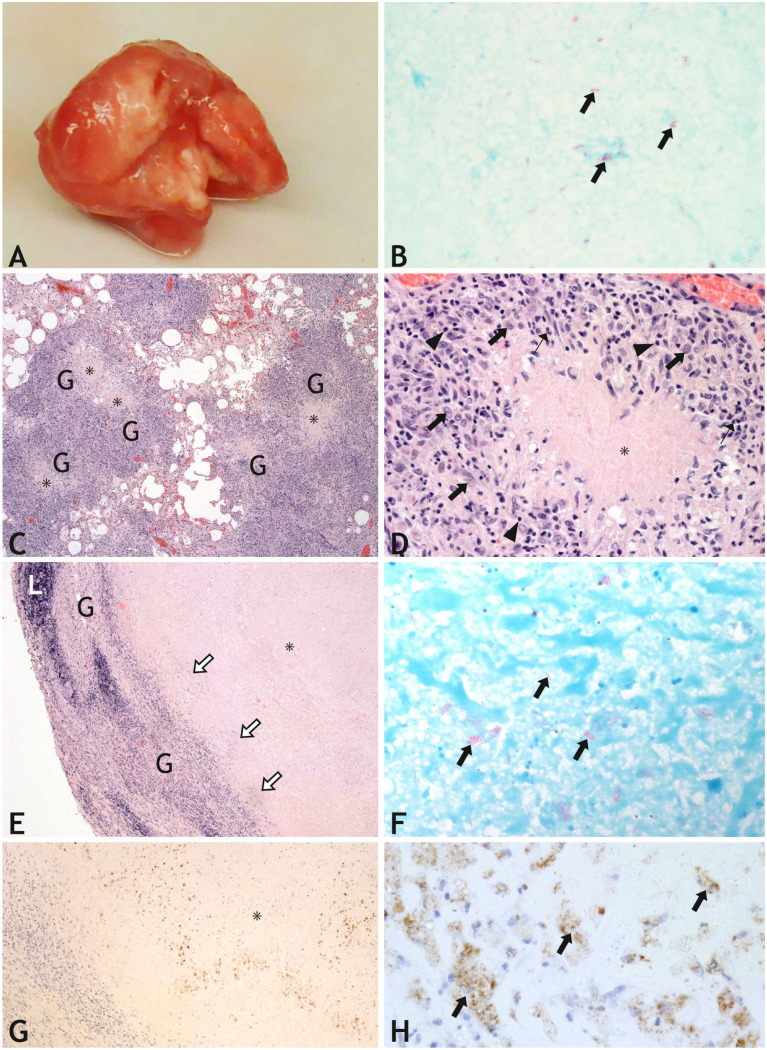
Tuberculosis in cat 4 in a German household. **A.** Tracheobronchial lymph node with small granulomas on the cut surface. **B.** Tissue imprint from the tracheobronchial lymph node on a glass slide, with acid-fast rods (arrows). Kinyon stain. **C.** Histology of the consolidated lung with confluent granulomas (G), most with areas of central necrosis (*). H&E. **D.** Granuloma with an area of central caseous necrosis (*), surrounded by a mixed infiltrate of epithelioid cells (thick arrows), macrophages, lymphocytes (arrowheads), and a few neutrophils (thin arrows). H&E. **E.** Part of a large granuloma with extensive necrosis (*) demarcated by a granulomatous infiltrate (G) in the tracheobronchial lymph node. At the edge of the necrotic area, groups of intact and necrotic neutrophils are present (white arrows). Only small remnants of lymphoid tissue (L) remain in the subcapsular region. H&E. **F.** Single and small groups of acid-fast rods (arrows) within the necrotic area. Ziehl–Neelsen stain. **G.** Immunohistochemistry reveals higher numbers of mycobacteria (brown), often in groups, irregularly distributed within the necrotic area (*). **H.** In higher magnification, many short rod-shaped bacteria (brown) associated with “ghost cells” (arrows) and nuclear debris within the necrotic areas are seen.

At autopsy, the cat was in good nutritional condition. The lungs were firm, nodular, and diffusely infiltrated by solid white material. The tracheobronchial lymph nodes were markedly enlarged and had white spots (**Fig. 4A**). The liver was moderately swollen, with pale lobule markings and fine white foci in the parenchyma. Acid-fast bacteria were found in tissue imprints of the lymph nodes (**Fig. 4B**).

Histologically, numerous coalescing granulomas (**Fig. 4C**) in the lung had large areas of central caseous necrosis with some nuclear debris and a few intact and degraded neutrophils at the periphery. The necrotic centers were surrounded by a variably wide zone of epithelioid cells and macrophages, as well as lymphocytes and a few plasma cells (**Fig. 4D**). A few bi- and multinucleate cells were present. The granulomas often extended to the pleura, resulting in granulomatous pleuritis. Many airways were filled with necrotic material and inflammatory exudate. Similar granulomas were found in the kidney and pancreas. Large amounts of necrotic material and inflammatory exudate were present in the renal pelves. Small granulomas were found in the spleen and liver. Neither bacteria nor fungi were detected by special stains, and immunohistochemistry for *M. bovis* was negative.

In the tracheobronchial lymph nodes, most of the lymphoid tissue was replaced by confluent granulomas with extensive areas of caseous necrosis (**Fig. 4E**). Within the necrotic centers of granulomas, a few small areas of mineralization and streaks of nuclear debris were present. At the periphery, single-to-small groups of intact and degraded neutrophils were present. Thick fibrous tissue was present in areas where the granulomas extended to the capsule of the lymph node. Ziehl–Neelsen staining revealed single and small groups of acid-fast rod bacteria within the necrotic areas (**Fig. 4F**). Larger numbers of mycobacteria were detected by IHC (**Fig. 4G**) and were unevenly distributed throughout the necrotic areas. Single and groups of rod-shaped bacteria were often associated with “ghost cells” and nuclear debris within the necrotic areas (**Fig. 4H**).

For direct rtPCR analysis of DNA extracts of the tissue and culture, lung, lymph node, liver, spleen, and kidney tissues were pooled and further processed. In the pooled sample, DNA specific for MTC bacteria was detected, and *M. bovis* was identified by culture.

### Epidemiologic investigations

#### Genomic analysis

Using the classical spoligotyping method, both isolates were identified as *M. bovis* spoligotype SB0120 (IT482). Finer genetic typing was performed using PCR-based MIRU-VNTR analysis of 26 different loci. The MIRU-VNTR-pattern found (1-2-6-3-2-2-3-3-2-2-6-3-4-3-4-2-3-3-3-3-2-4-1-2-10-5) was identical for the isolates from both cats and had not been reported previously in *M. bovis* isolates from animals in Germany. Analysis of the genome sequences with TB profiler confirmed the spoligotype SB0120 (IT482), additionally revealed lineage La1.4, and detected no resistance determinants against commonly used anti-tuberculosis drugs, aside from the characteristic *M. bovis* pyrazinamide resistance.

**Figure 5.**
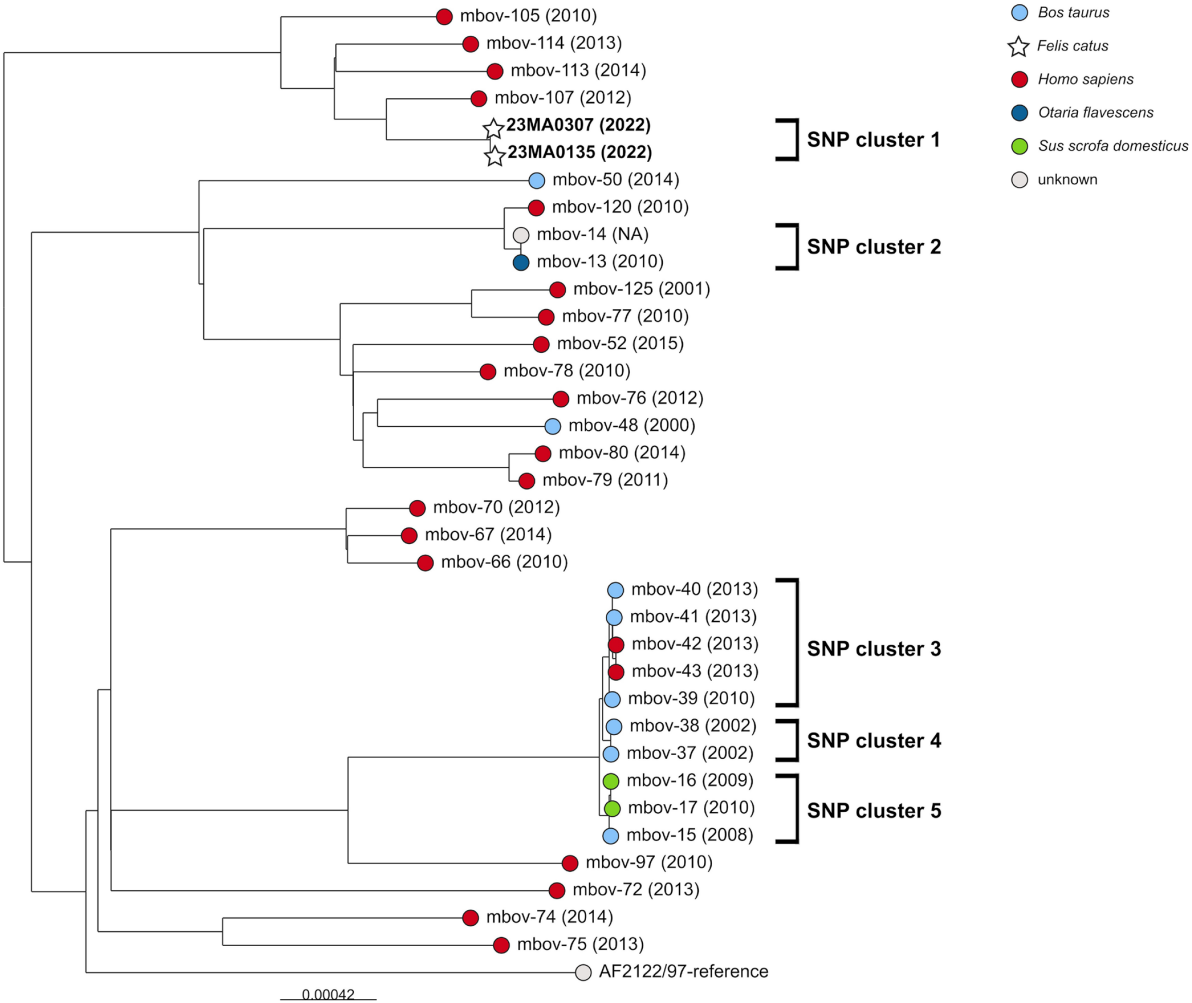
Feline tuberculosis in a German household. Midpoint-rooted maximum-likelihood tree based on cgSNP alignment of German *Mycobacterium bovis* spoligotype SB0120 isolates (data from Kohl, Kranzer,^28^ and our study). The leaves are colored according to the host. Scale bar = the nucleotide changes per base position.

The isolates from both cats were identical in single-nucleotide polymorphism (SNP) analysis of the WGS data, with only a single cgSNP different. To test if the current isolates were genetically close to previously isolated German *M. bovis* spoligotype SB0120 strains, other WGS data,^28^ which also comprised animal isolates, were included in the analysis. The data of 2 strains from that study^28^ were excluded after preliminary analysis because of quality issues. In total, 3,756 core genome SNPs were called. In the midpoint-rooted maximum likelihood tree (**Fig. 5**), the isolates from the 2 cats formed a cluster (SNP cluster 1) separate from human isolates. The SNP distance to the closest match (mbov-107) was 120 and 121 SNPs, respectively, for the 2 feline isolates. When applying a 12 SNP threshold^28^ to define an epidemiologically linked outbreak, 5 different clusters of isolates could be distinguished, which comprised strains isolated from different hosts (e.g., cattle and human [SNP cluster 3] or cattle and domestic pig [SNP cluster 5]).

#### Serology

Cat 4 serum was strongly ELISA-positive (value >4 in the original 1:25 dilution). Performing log_10_ dilution steps, cat 4 still tested positive at a 1:2,500 dilution. In contrast, cats 5–7 were negative in ELISA testing, which remained the case 4 mo later when the animals were re-tested.

#### Zoonotic impact

Both owners as well as the local veterinarians were asked by the local health agency to undergo an interferon-gamma release assay (IGRA). Only the 2 owners accepted the offer to test, and 1 of them was positive (data not shown). Radiography of the positive owner did not reveal any suspicious lung changes; therefore, latent TB was diagnosed. The patient was still in a TB monitoring program at the last contact made in December 2023.

## Discussion

*M. bovis* has the widest known host range of all MTC members. In humans, ~150,000 new infections and 12,500 deaths caused by *M. bovis* are estimated each year worldwide.^58^ However, the most important hosts for this pathogen are cattle. Bovine tuberculosis (**bTB**) has a large economic impact in low and middle-income countries. In contrast, in the European Union (EU), several countries (notably in central and northern Europe) have been declared OTF from bTB as a result of strict eradication programs over the last century. Germany achieved the OTF status in 1997, meaning that <0.1% of the German cattle herds are affected by bTB. The EU lists infections with *M. bovis*, *M. caprae*, and *M. tuberculosis* in cattle as category B diseases and has the goal to eradicate them. In other artiodactyls (category D), surveillance programs are mandatory before animals are allowed to be moved to or within the EU. In the remaining terrestrial mammals (category E), infections must only be monitored.

Contrary to cattle, the tools for antemortem diagnosis of TB in pet animals are limited. In cattle, the diagnosis of bTB is based on the measurement of cell-mediated immunity using intradermal tuberculin skin tests with bovine purified protein derivates (**PPD_bov_**), alone or in combination with avian PPD, or as an in vitro IGRA assay by stimulating blood cells with the respective PPDs. However, skin tests with *M. bovis*–sensitized cats had only a low positive predictive value, given that the skin fold increased only very moderately (≤0.3 mm) 72 h after PPD_bov_ application compared with control cats (≤0.05 mm).^17^ In the United Kingdom, feline IGRA assays were developed and showed good performance in predicting *M. bovis* or *M. microti* infections in cats.^42^ Unfortunately, this assay was not available for our study. For testing the humoral immune response against *M. bovis* infections in cats, several different antigens have been investigated.^17,41,43^ In *M. bovis–*infected cats, the MPB83 antigen was the most reactive antigen^17,43^ and is also the test antigen in the ELISA that we used in our study (resulting in a strong positive result for cat 4). Even though only limited data on serology in cats are available, MPB83-based serology might be helpful in antemortem testing and/or surveillance of contact animals.

In the early stages of infection, clinical signs of TB may be inapparent. As disease progresses, nonspecific signs (e.g., reduced body condition, cachexia, lymphadenopathy, or respiratory disorders) can occur.^35^ Depending on the affected organ system, cats may be blind^51^ or lame.^31^ Results of analysis of blood measurands (CBC, biochemistry) are mostly nonspecific. Sometimes, a shift in the albumin:globulin ratio caused by decreased albumin and increased globulin concentrations is apparent in animals with mycobacteriosis.^26,47^ We did note this change in cat 3. Radiography or computed tomography may be informative in detecting abnormalities not discernible on physical examination (e.g., disseminated lymphadenopathies or consolidated lung parenchyma).^24^ In our cases, radiographic findings included severely consolidated lung tissue in cat 3 and consolidation of the left cranial lung field in cat 4.

Although many publications have described one or more enlarged lymph nodes as prominent pathology findings,^1,13,15,21,44^ we found an enlarged tracheobronchial lymph node only in cat 4. Furthermore, although both animals had firm, consolidated lungs at autopsy, pathognomonic granulomas were only macroscopically visible in the lung, tracheobronchial lymph nodes, and liver of cat 4. Thus, for accurate diagnosis, inclusion of microbiologic identification of the causative agent as a part of the postmortem examination should be considered the gold standard.

In cat 3, lesions were detected only at the primary site of infection. This is characteristic of the post-primary phase of infection, resulting in chronic organ TB. In cat 4, lung, bronchial lymph node, and multiple other organs were affected. Necrotic material and inflammatory cells from granulomas were present within airways, indicating a post-primary phase infection. Dissemination to other organs is indicative of late generalization, which occurs by lympho-hematogenous spread. These lesions are, as seen in cat 4, poorly mineralized. They may occur in anergic animals.

Differences in the morphology of the granulomas between the 2 cats detected by histologic examinations may be the result of differences in the duration of infection (thus, stage of TB) or differences in the individual immune response. In agreement with other authors, multinucleate giant cells of the Langhans type were not seen in granulomas in either cat.^13,22,32,33^ As also commonly described for *M. bovis*, bacteria were rarely detected in lesions. Acid-fast staining and IHC results were congruent, which indicates that this lack of detection was not based on changes in the mycobacterial cell wall resulting in different staining properties. Mycobacteria were detected only in the tracheobronchial lymph node of cat 4, which was the organ with the most extensive necrosis. IHC allowed easier detection and revealed larger numbers of bacteria compared with Ziehl–Neelsen staining. Detection by microscopy, as well as by cultivation and molecular genetic analysis, were further complicated by the lack of even distribution of *M. bovis* within the necrotic tissue.

Genetic characterization of both strains revealed a common spoligotype, SB0120, which is frequently detected in cattle and humans in several European and African countries.^7,25,28,29,45^ Strains with this spoligotype have also been isolated from cattle and humans in Turkey.^23,54^ Comparing the 4 MIRU-VNTR profiles among 9 *M. bovis* isolates from cattle,^23^ we found at least 5 differing loci in 24 loci comparable to the strains from our study. Unfortunately, WGS data were not available from *M. bovis* spoligotype SB0120 isolates from Turkey. Therefore, we compared the data with published German *M. bovis* spoligotype SB0120 isolates of human or animal origin. Both of our feline *M. bovis* spoligotype SB0120 isolates clearly separated from the German isolates, with a minimum difference of 120 SNPs. For *M. tuberculosis*, the mutation rate was estimated between 0.5 and 1 SNPs/y,^56^ highlighting the large genetic distance between these human and animal German isolates and the feline ones from our study. Nevertheless, regarding the high diversity of *M. bovis* spoligotype SB0120 in Germany, the source of the cats’ infection cannot be determined unambiguously, based on the available genomic data.

Considering the long incubation time typical for TB, it is likely that the animals may have been infected before they were brought to Germany. In OTF countries, the awareness for TB is low, especially when animal species other than cattle are affected. In 2025, several colleagues published feline TB case studies (e.g., *M. bovis* infections in Argentina^5^ and Brazil,^15^ *M. africanum* infection in Italy,^1^ *M. bovis* infections in the Netherlands^13^), reflecting an increased alertness for this disease in pet animals. Although no mandatory national control measures exist for TB in cats, the detection of a positive TB test in owners shows that the zoonotic risk should not be underestimated.

## Conclusion for practice

- Importing animals from certain regions and countries carries the risk of introducing diseases that occur only rarely or not at all in Germany.
- TB should be included as a differential diagnosis in cats with respiratory disorders or cutaneous lesions, especially if the animals originate from countries with higher TB prevalence.
- For diagnosis in animals with no obvious tissue lesions, the analysis of multiple organs might increase the sensitivity of detection, given that the distribution of the pathogen in tissues is not homogeneous.
- Intense testing is needed to estimate the zoonotic risk for humans in close contact with infected animals.

Our case series highlights the problem of inadequate TB test procedures in pet animals. First, antemortem, when even limited test options are not fully available or the available tests are not utilized; and second, postmortem, when owners—for financial, ethical, or emotional reasons—do not have their animals autopsied after death.
